# Omega 3 fatty acids preserve testicular function by ameliorating BPF-induced dysthyroidism: role of p53/Bcl-2 signaling and proton pump activities

**DOI:** 10.5935/1518-0557.20240033

**Published:** 2024

**Authors:** Adeyemi Fatai Odetayo, Luqman Aribidesi Olayaki

**Affiliations:** 1 Department of Physiology, University of Ilorin, Ilorin, Nigeria; 2 Department of Physiology, Federal University of Health Sciences, Ila Orangun, Nigeria

**Keywords:** apoptosis, bisphenol F, hypothyroidism, omega 3 fatty acid, p53/BCl-2 signaling, proton pump

## Abstract

**Objective:**

Bisphenol F (BPF) is an endocrinedisrupting chemical, but information about its effect on thyroid hormones has not been fully explored. Omega 3 fatty acids (O3FA), on the other hand, are antioxidant and antiapoptotic agents. Therefore, this study explored the role and associated molecular mechanism of O3FA in BPF-induced hypothyroidism-mediated testicular dysfunction in male Wistar rats.

**Methods:**

Twenty (20) male Wistar rats were randomized into four groups (n=5/group), namely: the control group; the BPF treated group (30 mg/kg of BPF); and the intervention groups (30mg/kg BPF + 100mg/kg O3FA (BPF+O3FA-L) and 30mg/kg BPF + 300mg/kg of O3FA for 28 days).

**Results:**

Low and high doses of O3FA ameliorated BPF-induced hypothyroidism-mediated reduction in sperm quality, testosterone, luteinizing hormone, follicle-stimulating hormone, catalase, superoxide dismutase, total antioxidant capacity, and nuclear factor erythroid 2-related factor 2 and increases in estrogen, malondialdehyde, c-reactive protein, interleukin 1 beta, caspase 3. Furthermore, O3FA prevented BPF-induced Na+/K+−ATPase and Ca2+−ATPase dysfunction, estrogen receptor beta overexpression, and tumor protein P53 (p53)/ b-cell lymphoma 2 (Bcl-2) imbalance.

**Conclusions:**

This study showed that O3FA ameliorated BPF-induced dysthyroidism-mediated testicular dysfunction by preventing proton pump dysfunction and p53/BCl-2 imbalance.

## INTRODUCTION

Bisphenol A (BPA) and its analogs, such as bisphenol F (BPF), are raw materials widely used in the food, pharmaceutical, chemical, and canning industries. BPA is the most widely used bisphenol, which has been implicated in various human diseases, such as metabolic syndrome (Caporossi & Papaleo, 2017). Thus, many countries have restricted BPA usage (Fatai & Aribidesi, 2022). Alternatively, BPF, considered the major replacement for BPA, has been introduced in the industries.

With the increasing usage, BPF occurrence in the environment is rising. In addition to its presence in food and medical devices, BPF has also been found in environmental media, such as water, dust, thermal paper, and water (Yuan *et al.,* 2019). Despite the increasing usage of BPF, its gonadotoxic effect remains underexplored. Previous findings have shown that BPF impaired testicular function by disrupting the hypothalamic-pituitary-gonadal (HPG)-axis (Fatai & Aribidesi, 2022), redox balance (Ullah *et al.,* 2019; Odetayo & Olayaki, 2022), and apoptotic markers activities (Ferreira *et al.,* 2022; Odetayo *et al.,* 2023a). Despite these findings, the effect of BPF on the thyroid gland has not been fully explored, nor has it been established whether BPF-induced testicular dysfunction is associated by with dysthyroidism.

Thyroid hormone (TH) has important roles in growth, oxygen consumption regulation, mitochondrial energy metabolism, and other biological processes (Meng *et al.,* 2016). TH has also been described as one of the major hormones responsible for testicular maturation and growth (Sahoo *et al.,* 2008), with dysthyroidism linked with testicular dysfunction (El-Kashlan *et al.,* 2015). The presence of thyroid receptors (TRs) and thyroid hormone transporters in the Sertoli, germ, and Leydig cells further substantiates the role of TH in testicular function (Gao *et al.,* 2014). Aside from the presence of TRs, these testicular cells also contain deiodinases, responsible for converting thyroxine (T4) into active triiodothyronine (T3) and vice versa. Hence, testicular cells are equipped with the transporters and enzymes required to maintain thyroid hormone homeostasis within the testes (Gao *et al.,* 2014). Physiologically, TH is an important factor in redox balance, and alteration in thyroid homeostasis can lead to oxidative stress, apoptosis, and proton pump dysfunction (Chang *et al.,* 2019). Hypothyroidism has been shown to disrupt proton pump (such as Ca2+-ATPase) activities, which in turn disrupts calcium homeostasis (Chang *et al.,* 2019). Maintaining intracellular calcium is important for mitochondrial function since calcium imbalance is a major trigger for mitochondrial dysfunction associated with oxidative stress and apoptosis (Nazıroğlu *et al.,* 2012).

Hence, it is plausible to predict that BPF-impaired testicular function might be associated with thyroid dyshomeostasis and proton pump dysfunction since BPF has been linked with testicular oxidative damage and apoptosis (Odetayo *et al.,* 2023b).

Despite the available information on dysthyroidism and oxidative stress, the role of thyroid homeostasis in apoptosis has not been fully explored. Although hypothyroidism has been associated with increased apoptotic markers (Mukherjee *et al.,* 2014), the role of tumor protein P53 (p53)/ b-cell lymphoma 2 (Bcl-2) signaling has not been explored. P53 signaling is responsible for regulating different cell reactions related to apoptosis. It is a core participant in apoptosis regulation by stimulating the caspase-dependent pathway via the modulation of multiple apoptotic markers such as BCl-2 (Muller & Vousden, 2013). Thus, P53 directly or indirectly regulates apoptosis at different levels (Moulder *et al.,* 2018).

On the other hand, omega 3 fatty acids (O3FA) are antioxidants that can be obtained from diet. O3FA are polyunsaturated fatty acids (PUFA) with established protective roles in the cardiovascular system (Jain *et al.,* 2015), liver (Parker *et al.,* 2012), kidney (Hu *et al.,* 2017), and testes (Akhigbe *et al.,* 2021a; Odetayo & Olayaki, 2023). In addition, O3FA are antioxidant and anti-inflammatory agents considered the precursors of key active metabolites for treating several diseases (Abdel-Baky *et al.,* 2023). This information suggests that O3FA might be a promising supplement for protecting cells from extrinsic toxic stimuli such as BPF.

Despite these pieces of information, no study has explored the possible role of TH homeostasis on BPF-induced testicular dysfunction or the role of proton pump activity on BPF-induced gonadotoxicity. Although O3FA are anti-apoptotic agents, the role of P53/BCl-2 signaling in O3FA-mediated anti-apoptotic effect has not been explored. Hence, this study was designed to establish the effect of dysthyroidism in BPF-induced testicular dysfunction and the possible ameliorative effect of O3FA. Additionally, the roles of proton pumps and P53/BCl-2 signaling as the possible mechanism of action were explored.

## MATERIALS AND METHODS

### Chemicals

Each capsule of O3FA used in this study contained eicosapentaenoic acid (EPA) and docosahexaenoic acid (DHA) at a ratio of 3:2. The capsules were procured from Gujarat Liqui Pharmacaps Pvt. Ltd. Vadodara, Gujarat, India. All other chemicals except otherwise stated were purchased from Sigma Aldrich.

### Animals

The twenty male Wistar rats (age: 10-13 weeks, weights: 160-180g) obtained from the Biochemistry Department of the University of Ilorin were randomized into four groups (n=5) after two weeks of acclimatization. The animals in Group 1 (Control) were treated with 0.5 ml of corn oil; Group 2 (BPF) received 30 mg/kg of BPF; Group 3 (BPF+O3FA-L) and Group 4 (BPF+O3A-H) received BPF + low (100 mg/kg) and high (300 mg/kg) doses of O3FA respectively. The BPF dosage used in this study was similar to the doses previously reported by Higashihara *et al.* (2007), Ullah *et al.* (2019), and Fatai & Aribidesi (2022), while the dosage of O3FA was earlier reported and used by Adeyemi & Olayaki (2017).

The designed experimental protocol was approved by the University of Ilorin Review and Ethical Committee and followed the “National Institute of Health guidelines using the guide for the care and handling of laboratory animals (NIH Publication No. 80-23; amended 1978)”. The experimental protocol complied with the National Research Council’s guidelines for the Care and Use of Laboratory Animals and the ARRIVE guidelines for reporting experimental findings.

### Sample Collection

The calculated dosage of BPF for each animal was dissolved in corn oil so that each received 0.5 ml of the solution. The solution was administered orally to mimic the main route of BPF exposure. The rats were given the solution for 28 days, and the overnight fasted animals were sacrificed after 24 hours from the last treatment with intraperitoneal ketamine (40 mg/kg) and xylazine (4 mg/kg) (Afolabi *et al.,* 2022a). The blood samples obtained via cardiac puncture were centrifuged at 3000 rpm to obtain serum for hormonal assays. The left testes were homogenized in a cold phosphate buffer solution for biochemical analysis. The right testes were harvested for immunohis-tochemistry testing, while the epididymides were removed for sperm analysis.

### Sperm analysis

Each caudal epididymis was prepared in a clean petri dish, and sperm count, motility, and morphology were estimated according to previous methods (Akhigbe *et al.,* 2021b; Afolabi *et al.,* 2022b).

### Hormonal Assay

Serum T3, T4, thyroid stimulating hormone (TSH) (Carlbiotech, USA) and luteinizing hormone (LH), follicle-stimulating hormone (FSH), testosterone, and estradiol (Bio-Inteco, UK) were determined using ELISA kits according to the manufacturer’s guidelines.

### Oxidative stress, inflammatory, and apoptotic markers

Testicular malondialdehyde (MDA), superoxide dismutase (SOD), and catalase (CAT) were determined as previously established (Afolabi *et al.,* 2022c; Akhigbe *et al.,* 2023; Olayaki *et al.,* 2023). Total antioxidant capacity (TAC) (Fortress Diagnostic Kit, Switzerland) was estimated using a colorimetry method. Testicular interleukin-1 beta (IL-1P) (Nanjing Mornmed Medical, China), C-reactive protein (CRP), nuclear factor erythroid 2-related factor 2 (Nrf2), and caspase-3 (Elabscience, USA) were determined as described by the test kit manufacturers.

### Proton pump

Testicular transmembrane protein (Na+/K+−ATPase and Ca2+−ATPase) activity was determined spectrophotometrically using the method of Torlinska & Grochowalska (2004).

### Immunohistochemistry

Testicular estrogen receptor β (Erβ), P53, and BCl-2 were determined as previously described by Odetayo *et al.* (2023a). “Formalin-fixed and paraffin-embedded testicular tissues were sectioned at 4 µm for immunohistochemistry. Immunohistochemical procedures were performed using appropriate antibodies; anti-mouse Erβ monoclonal for Erβ expression (1:100), anti-mouse p53 monoclonal for p53 expression (1:100), and anti-mouse Bcl-2 monoclonal for Bcl-2 expression (1:200). The formalin-fixed and paraffin-embedded testicular tissues were sectioned at 4 µm for immunohistochemistry. Appropriate antibodies; anti-mouse Erβ monoclonal for Erβ expression (1:100) (Leica Biosystems, USA with CAT NO: 6069100), anti-mouse p53 monoclonal for p53 expression (1:100) (Espredia with CAT NO: 186P2105D), and anti-mouse Bcl-2 monoclonal for Bcl-2 expression (1:200) (Thermo Fisher Scientific, USA)”.

### Statistical analysis

Software package GraphPad PRISM 5 (GraphPad Software, La Jolla, California, USA) was used in statistical analysis with one-way analysis of variance (ANOVA) and Tukey’s post hoc test. Data were reported as mean ± standard deviation. Values of *p* below 0.05 were considered statistically significant.

## RESULTS

### Sperm quality

**Figure 1:**
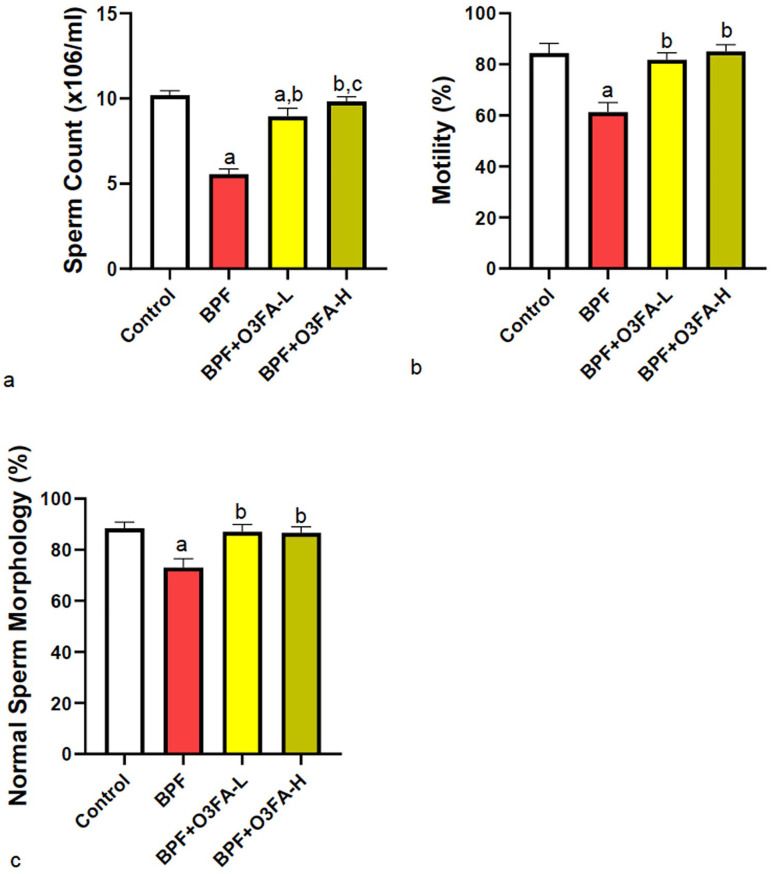
Effect of omega 3 fatty acids on (a) sperm count (b) motility (c) morphology in BPF-exposed rats. a. *p*<0 .05 versus control, b. *p*<0.05 versus BPF, c. *p*<0.05 versus BPF + O3FA-L using one-way analysis of variance (ANOVA) followed by Tukey’s post hoc test for pairwise comparison. BPF: Bisphenol F, O3FA-L: omega-3 fatty acid low dose, O3FA-H: omega-3 fatty acid high dose.

Low (O3FA-L) and high doses (OFA3-H) of O3FA ameliorated BPF-induced decrease in sperm count, motility, and morphology compared with controls (Figure 1). Although there was no significant difference in sperm motility and morphology in the rats given O3FA-L and OFA3-H, the animals treated with a high dose of O3FA had better sperm counts than their counterparts treated with a low dose.

### Hormones

**Figure 2:**
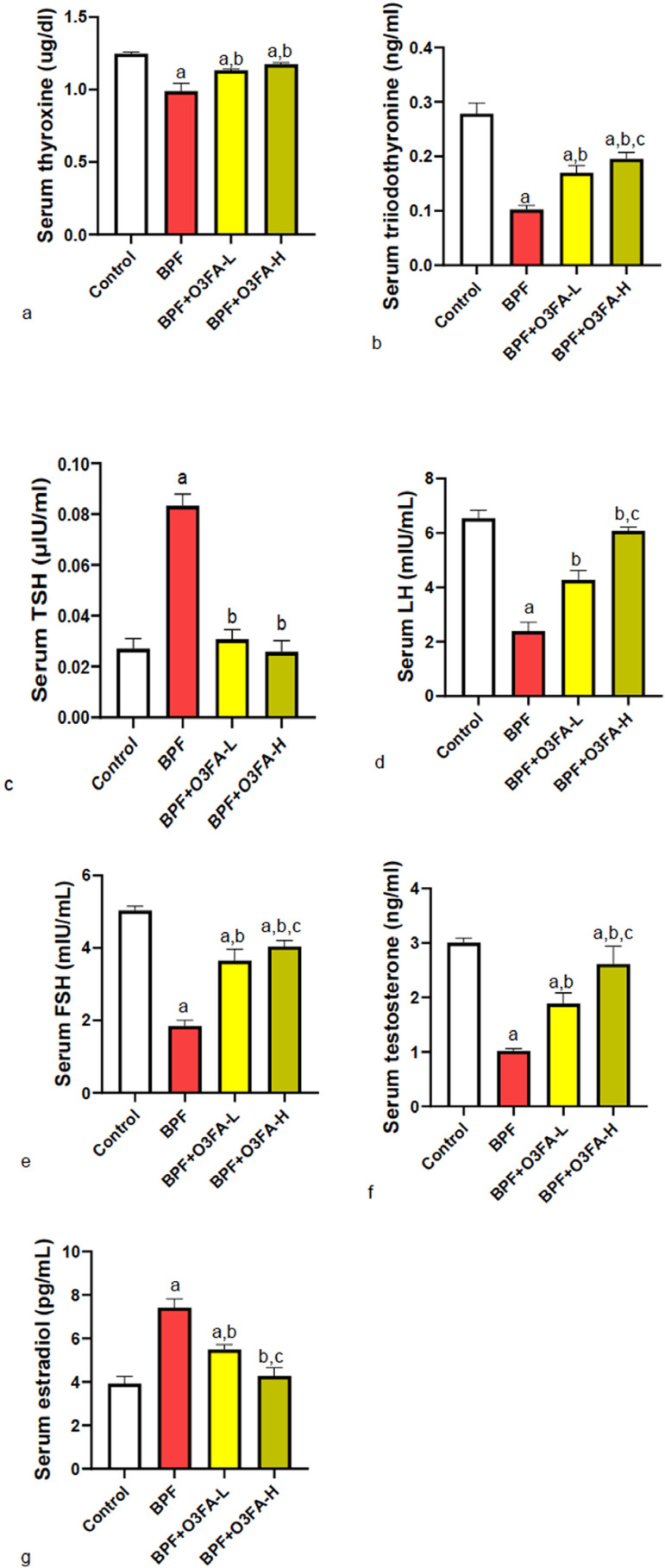
Effect of omega 3 fatty acids on serum (a) thyroxine (b) triiodothyronine (c) thyroid stimulating hormone (TSH) (d) luteinizing hormone (LH) (e) follicle stimulating hormone (FSH) (f) testosterone (g) estradiol in BPF-exposed rats. a. *p*<0.05 versus control, b. *p*<0.05 versus BPF, c. *p*<0.05 versus BPF + O3FA-L using oneway analysis of variance (ANOVA) followed by Tukey’s post hoc test for pairwise comparison. BPF: Bisphenol F, O3FA-L: omega-3 fatty acid low dose, O3FA-H: omega-3 fatty acid high dose.

BPF disrupted thyroid homeostasis, as evidenced by the significant decrease in T4 and T3 and the increase in TSH compared with controls (Figure 2). The observed alterations were prevented by the co-treatment of BPF with low and high doses of O3FA. However, animals treated with O3FA-H had better circulatory T3 levels than those treated with O3FA-L. Furthermore, the observed decrease in serum LH, FSH, and testosterone and increase in estradiol following BPF treatment compared with controls were blunted by both doses of O3FA. Although both doses of O3FA prevented the observed reproductive hormonal imbalance, the ameliorative effect was more pronounced in animals treated with high doses.

### Oxidative stress

**Figure 3:**
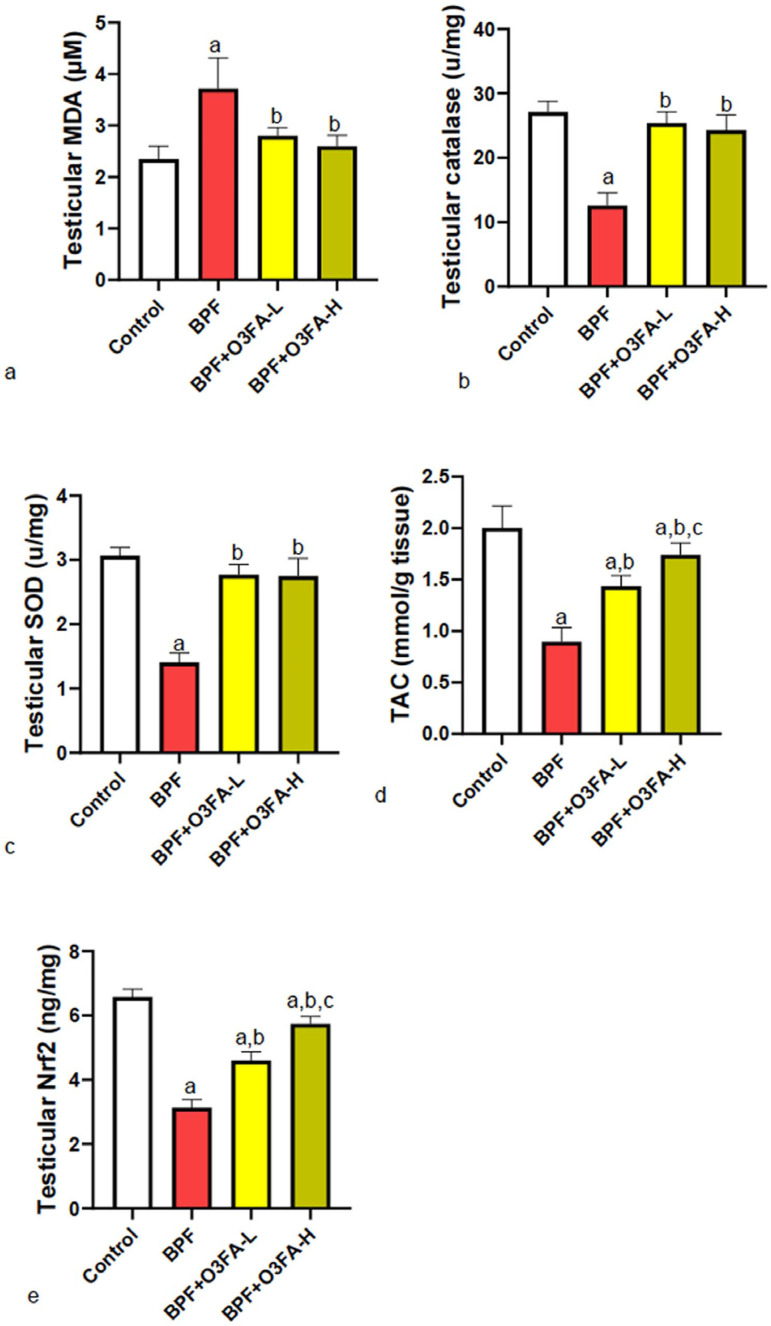
Effect of omega 3 fatty acids on testicular (a) malondialdehyde (MDA) (b) catalase (CAT) (c) superoxide dismutase (SOD) (d) total antioxidant capacity (TAC) (e) nuclear factor erythroid 2-related factor 2 (nrf2) in BPF-exposed rats. a. *p*<0.05 *versus* control, b. *p*<0.05 *versus* BPF, c. *p*<0.05 versus BPF + O3FA-L using oneway analysis of variance (ANOVA) followed by Tukey’s post hoc test for pairwise comparison. BPF: Bisphenol F, O3FA-L: omega-3 fatty acid low dose, O3FA-H: omega-3 fatty acid high dose.

BPF exposure led to a significant increase in testicular MDA and a decrease in CAT, SOD, TAC, and Nrf2 compared with controls (Figure 3). Both doses of O3FA ameliorated the redox imbalance, although animals treated with O3FA-H were significantly different in TAC and Nrf2 levels compared with those treated with O3FA-L.

### Inflammatory and Apoptotic markers

**Figure 4:**
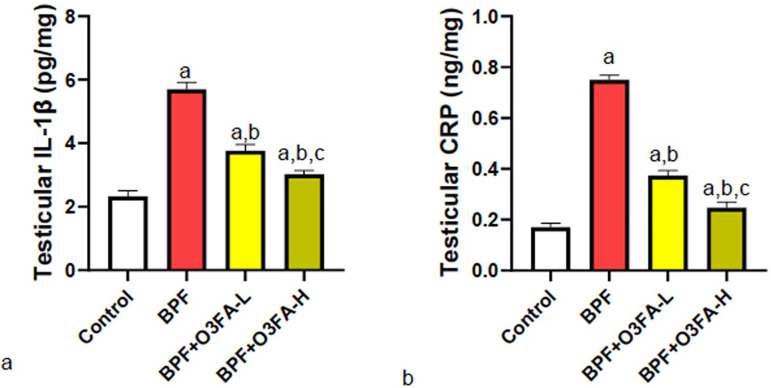
Effect of omega 3 fatty acids on testicular (a) interleukin 1 beta (IL-1β) (b) c-reactive protein (CRP) in BPF-exposed rats. a. *p*<0.05 versus control, b. *p*<0.05 versus BPF, c. *p*<0.05 versus BPF + O3FA-L using one-way analysis of variance (ANOVA) followed by Tukey’s post hoc test for pairwise comparison. BPF: Bisphenol F, O3FA-L: omega-3 fatty acid low dose, O3FA-H: omega-3 fatty acid high dose.

**Figure 5:**
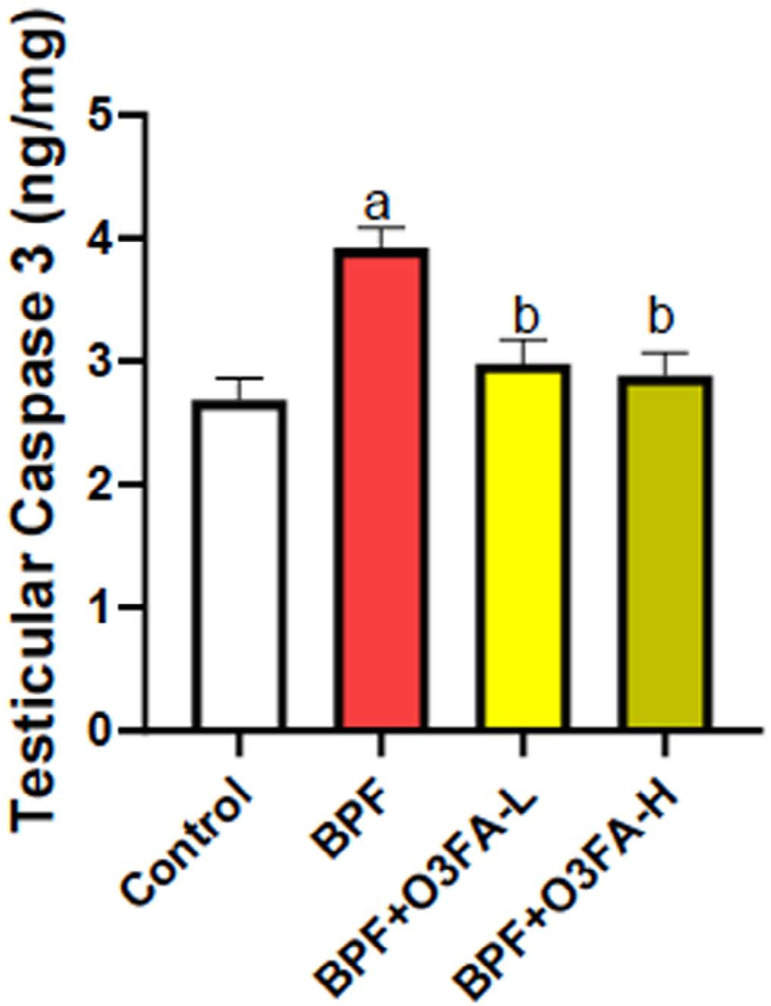
Effect of omega 3 fatty acids on testicular caspase 3 in BPF-exposed rats. a. *p*<0.05 versus control, b. *p*<0.05 versus BPF, c. *p*<0.05 versus BPF + O3FA-L using one-way analysis of variance (ANOVA) followed by Tukey’s post hoc test for pairwise comparison. BPF: Bisphenol F, O3FA-L: omega-3 fatty acid low dose, O3FA-H: omega-3 fatty acid high dose.

O3FA prevented BPF-induced increase in testicular IL-1β, CRP, and caspase 3 compared with controls (Figures 4 and 5), although except for caspase 3, animals treated with a high dose of O3FA exhibited a better ameliorative effect in testicular parameters.

### Proton pump

**Figure 6:**
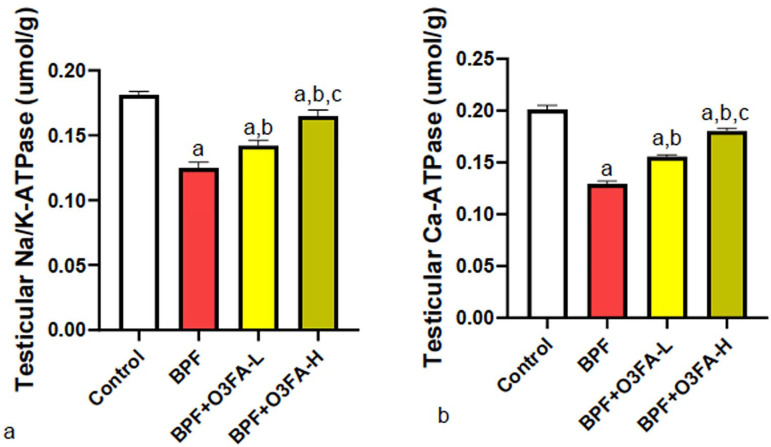
Effect of omega 3 fatty acids on testicular (a) sodium-potassium ATPase (Na-K ATPase) (b) calcium ATPase (Ca-ATPase) in BPF-exposed rats. a. *p*<0.05 *versus* control, b. *p*<0.05 *versus* BPF, c. *p*<0.05 *versus* BPF + O3FA-L using one-way analysis of variance (ANOVA) followed by Tukey’s post hoc test for pairwise comparison. BPF: Bisphenol F, O3FA-L: omega-3 fatty acid low dose, O3FA-H: omega-3 fatty acid high dose.

BPF affected proton pump activity compared with controls (Figure 6). While both doses of O3FA prevented the observed proton pump Na+−K+ ATPase and Ca2+ ATPase dysfunction, animals treated with high doses of O3FA exhibited a better ameliorative effect than their counterparts treated with a low dose.

### Er-β and P53/BCI-2 signaling

**Figure 7:**
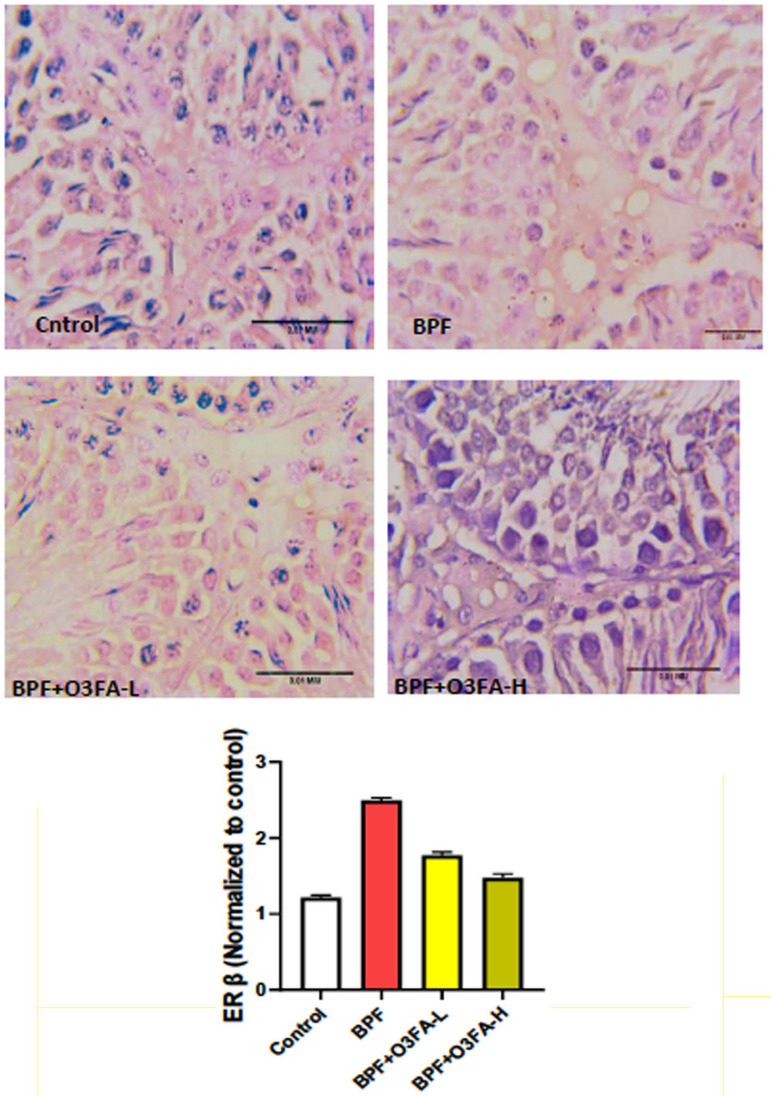
Effect of omega 3 fatty acids on testicular estrogen receptor beta (ER β) in BPF-exposed rats. a. *p*<0.05 *versus* control, b. *p*<0.05 *versus* BPF, c. *p*<0.05 *versus* BPF + O3FA-L using one-way analysis of variance (ANOVA) followed by Tukey’s post hoc test for pairwise comparison. BPF: Bisphenol F, O3FA-L: omega-3 fatty acid low dose, O3FA-H: omega-3 fatty acid high dose.

**Figure 8:**
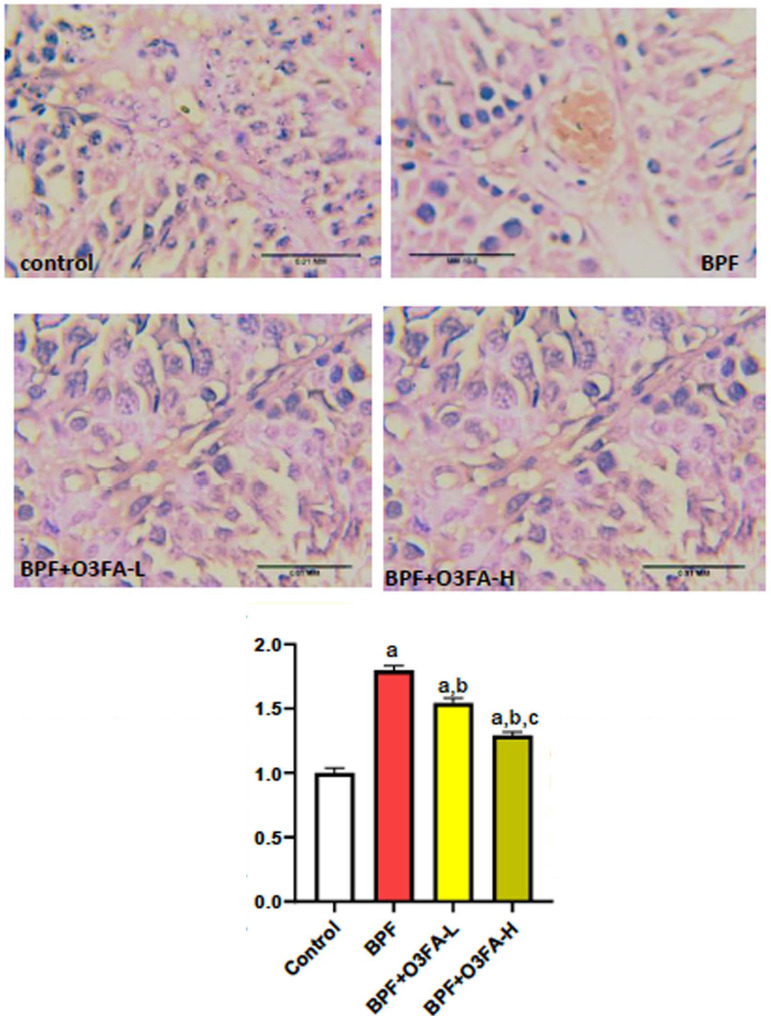
Effect of omega 3 fatty acids on testicular tumor protein p53 (p53) in BPF-exposed rats. a. *p*<0.05 *versus* control, b. *p*<0.05 *versus* BPF, c. *p*<0.05 *versus* BPF + O3FA-L using one-way analysis of variance (ANOVA) followed by Tukey’s post hoc test for pairwise comparison. BPF: Bisphenol F, O3FA-L: omega-3 fatty acid low dose, O3FA-H: omega-3 fatty acid high dose.

**Figure 9:**
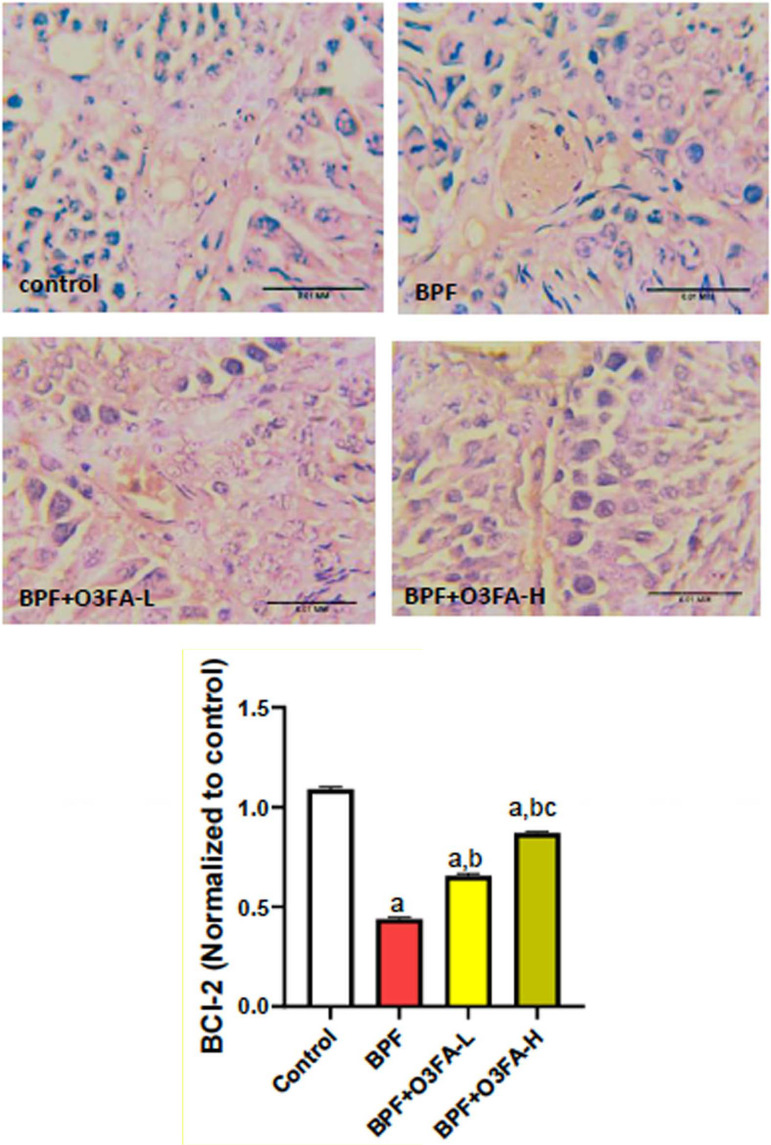
Effect of omega 3 fatty acids on testicular B-cell lymphoma 2 (BCl-2) in BPF-exposed rats. a. *p*<0.05 *versus* control, b. *p*<0.05 *versus* BPF, c. *p*<0.05 *versus* BPF + O3FA-L using one-way analysis of variance (ANOVA) followed by Tukey’s post hoc test for pairwise comparison. BPF: Bisphenol F, O3FA-L: omega-3 fatty acid low dose, O3FA-H: omega-3 fatty acid high dose.

Low and high doses of O3FA blunted the observed increase in Er-β and P53 and decreased BCL-2 following BPF exposure (Figures 7, 8, and 9). However, these ameliorative effects were more pronounced in animals treated with high doses of O3FA.

## DISCUSSION

The present study explored the effects of BPF on thyroid and testicular function. It also determined the role of Na+−K+ ATPase and Ca2+ ATPase activities and P53/BCl-2 signaling in testicular function. A possible ameliorative effect of O3FA on BPF-induced hypothyroidism was also established. It further hypothesized that BPF-induced testicular dysfunction was due to dysthyroidism-mediated Na+−K+ and Ca2+ ATPase dysfunction and P53/BCl-2 imbalance and that O3FA might ameliorate BPF-induced dysthyroidism. Our findings confirmed that BPF-induced hypothyroidism was associated with impaired testicular function. They also established that O3FA ameliorated BPF-induced hypothyroidism, oxidative stress, inflammatory response, and apoptosis in testicular tissues, as evidenced by the significant decreases in testicular CAT, SOD, and TAC, Nrf2, and the increases in MDA, IL-1β, CRP, and caspase 3. These events were accompanied by Na+−K+ and Ca2+ ATPase dysfunction and P53/BCl-2 imbalance in testicular tissues. In sum, our data suggest that O3FA restored testicular function by preventing BPF-induced hypothyroidism via Na+−K+ and Ca2+ ATPase and P53/BCl-2 mediated oxidative stress and apoptosis.

As expected, the results from this study showed that hypothyroidism (decreased T4 and T3 levels) was associated with testicular dysfunction, as evidenced by a significant decline in sperm quality and reproductive hormones. This confirms previous findings (Mazzilli *et al.,* 2023; El-Kashlan *et al.,* 2015; La Vignera *et al.,* 2017) demonstrating testicular dysfunction and male infertility in hypothyroidism. Hyperprolactinemia might be the link between the observed primary hypothyroidism and hypogonadotropic hypogonadism (Brown *et al.,* 2023) since BPF has been shown to increase circulatory prolactin (Odetayo & Olayaki, 2023). Hypothyroidism is associated with an increase in circulatory TRH, which in turn increases circulatory prolactin (Tashjian Jr *et al.,* 1971). This hypothyroidism-mediated hyperprolactinemia might directly impair the HPG-axis by inhibiting GnRH (Brown *et al.,* 2019) and LH (Gregory *et al.,* 2004) secretion, leading to a decline in circulatory testosterone. Furthermore, the findings from this study that BPF-induced hypothyroidism was associated with testicular oxidative damage, inflammation, and apoptosis aligns with the study of Kochman *et al.* (2021) and Bowman-Colin *et al.* (2016), which reported similar findings in dysthyroidism.

Although compelling evidence established that hypothyroidism induces testicular dysfunction, information about the associated mechanisms is still lacking. Therefore, the finding that BPF-induced hypothyroidism is associated with testicular transmembrane protein dysfunction is noteworthy.

BPF-induced testicular injury may be a consequence of dysthyroidism-induced testicular Na+/K+−ATPase and Ca2+−ATPase dysfunction (Chang *et al.,* 2019). Impairment of these transmembrane proteins depresses electrochemical gradient generation and maintenance across the testicular cell membrane and key organelles such as the mitochondria (Therien *et al.,* 1997). Additionally, Ca2+−ATPase maintains calcium homeostasis, which plays a key role in male fertility. The prostate gland (the main source of calcium for human semen), epididymis, and seminal vesicles require optimal calcium levels (Valsa *et al.,* 2016). Impaired steroidogenesis, sperm motility, chemotaxis, capacitation, and acrosome reaction have been reported in hypocalcemia (Beigi Harchegani *et al.,* 2019; Naz *et al.,* 2022). Also, men with hypomotility show lower calcium levels in semen than those with typical motility (Naz *et al.,* 2022), illustrating the importance of calcium homeostasis in male reproduction. Ca2+−ATPase is a key factor for regulating calcium homeostasis, and its dysfunction has been reported in calcium overload (Chang *et al.,* 2019). The testicular Na+/K+−ATPase and Ca2+−ATPase dysfunction in this study might be a consequence of the observed BPF-induced oxidative stress or increase in serum estrogen and the subsequent ER3 overexpression. Thyroid hormones play a major role in the maintenance of reactive oxygen species (ROS) generation, and dysfunction has been reported to cause the overproduction of ROS, leading to oxidative stress (Chang *et al.,* 2019), which might impair proton pump function (Zaidi, 2010). Proton pump inhibition has been linked with increased circulatory estrogen (Ding *et al.,* 2023). Thus, the observed increase in estrogen and estrogen receptors observed in this study might result from the observed proton pump dysfunction. Interestingly enough, both oxidative stress and ER3 are triggers of apoptosis (Odetayo *et al.,* 2023a), which culminates in the stimulation of p53/BCl-2-mediated apoptosis.

p53 is a key protein that regulates apoptosis via direct induction of Bax transcription, which can, in turn, overwhelm the antiapoptotic effects of BCl-2 (McCurrach *et al.,* 1997; Giorgi *et al.,* 2015). The activation of Bax leads to the release of mitochondrial cytochrome c, leading to the activation of caspase 3-mediated apoptosis (Schuler *et al.,* 2000). In addition, p53 can directly inhibit Bcl-2 activities by mimicking the proapoptotic ‘BH3-only’ class of Bcl-2 to bring about mitochondria permeabilization and apoptosis (Hemann & Lowe, 2006). Again, p53-induced PUMA protein can distort cytosolic p53-Bcl-2 complexes, eventually leading to the direct induction of p53-mediated apoptosis in mitochondria (Chipuk *et al.,* 2005). Furthermore, p53 can directly disrupt proton pump activity in the endoplasmic reticulum, leading to calcium overload and increased transfer to the mitochondria, leading to apoptosis induction (Giorgi *et al.,* 2015). Hence, the increase in p53 expression observed in this study might also explain the proton pump dysfunction following BPF-induced hypothyroidism. Our findings that BPF-induced hypothyroidism disrupted p53/BCl-2 signaling agreed with the study of Singh *et al.* (2003), in which increased apoptosis following a decrease in thyroid hormones was described.

Another important finding is the protective role of O3FA against BPF-induced hypothyroidism. This study revealed that O3FA prevented BPF-induced hypothyroidism-mediated testicular injury by restoring hormonal and redox balance and suppressing inflammatory and apoptotic markers, thus improving sperm parameters and testosterone synthesis. Although this is the first study to demonstrate the ameliorative effect of O3FA on BPF-induced hypothyroidism, our findings agreed with those of previous studies that described the antioxidant (Meital *et al.,* 2019), anti-inflammatory (Calder, 2017), antiapoptotic (Wendel & Heller, 2009), and thyroid protective (Abd Allah *et al.,* 2014; Benvenga *et al.,* 2022) effects of O3FA. In addition, O3FA prevents calcium overload during ischemic insult (Kromhout *et al.,* 2012), which further supports our claim that O3FA prevents BPF-induced dysthyroidism by preventing proton pump dysfunction.

## CONCLUSION

O3FA prevented BPF-induced primary (peripheral) hypothyroidism and secondary (hypogonadotropic) testicular dysfunction. O3FA also reversed the deleterious effects of BPF on testicular oxidative stress, inflammation, and apoptosis markers.
